# Multicenter prospective study on anastomotic leakage after right‐sided colon cancer surgery with laparoscopic intracorporeal overlap anastomosis (KYCC 2101)

**DOI:** 10.1002/ags3.12831

**Published:** 2024-06-05

**Authors:** Keisuke Kazama, Masakatsu Numata, Hiroyuki Mushiake, Nobuhiro Sugano, Teni Godai, Akio Higuchi, Tetsushi Ishiguro, Yosuke Atsumi, Satoru Shinoda, Aya Saito

**Affiliations:** ^1^ Department of Surgery Yokohama City University Yokohama Japan; ^2^ Department of Gastrointestinal Surgery Yokohama City University Medical Center Yokohama Japan; ^3^ Department of Surgery Saiseikai Yokohama City Nanbu Hospital Yokohama Japan; ^4^ Department of Surgery Hiratsuka Kyosai Hospital Hiratsuka Japan; ^5^ Department of Surgery Fujisawa Shounandai Hospital Fujisawa Japan; ^6^ Department of Surgery Yokohama Minami Kyosai Hospital Yokohama Japan; ^7^ Department of Biostatistics, School of Medicine Yokohama City University Yokohama Japan

**Keywords:** intracorporeal anastomosis, laparoscopic surgery, overlap anastomosis, prospective cohort study, right‐sided colon cancer

## Abstract

**Aim:**

Intracorporeal anastomosis (IA) is becoming increasingly popular and replacing extracorporeal anastomosis (EA) for reconstruction in laparoscopic and robotic surgery for right‐sided colon cancer (LSRCC). Intracorporeal overlap anastomosis (IOA) is the most widely used IA technique. This study aimed to examine the safety of IOA by investigating its short‐term results during the implementation phase.

**Methods:**

This multicenter prospective cohort study was conducted by the Kanagawa Yokohama Colorectal Cancer (KYCC) Study Group. Patients with stage 1–3 colon cancer who planned to undergo LSRCC with IOA reconstruction were eligible. The incidence of anastomotic leakage (AL) of Clavien–Dindo (C–D) grade ≥3 was evaluated as the primary endpoint, and other surgical outcomes and postoperative complications of C–D grades ≥2 were the secondary endpoints.

**Results:**

A total of 127 patients were enrolled, of whom 120 were finally analyzed. The incidence of C–D grade ≥2 complications was 8.3%. The incidence of C–D grade ≥3 AL was 0.8%. This trend was lower than that reported in previous randomized controlled trials (RCTs) and acceptable. Additionally, 1.7% of the patients developed abdominal abscesses, and no cases of anastomotic stenosis were observed. The median operative time was 257 min, and the reconstruction procedure required 32 min. Stapler closure of the enterotomy and facility experience of more than 30 cases were associated with a shorter reconstruction time during IOA.

**Conclusion:**

IOA is feasible and can be safely performed during the implementation phase in patients undergoing LSRCC.

## Graphical abstract

This prospective, observational study demonstrated the safety of overlap intracorporeal anastomosis for right‐sided colon cancer.

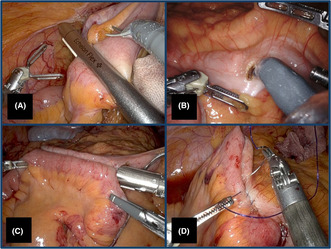

## 1 INTRODUCTION

Colorectal cancer (CRC) is one of the most common gastrointestinal cancers, and its incidence and mortality have increased worldwide.^1^ Despite the remarkable development of multimodal cancer therapies, primary cancer resection and lymph node dissection remain the key to achieving a cure for clinically nonmetastatic CRC.^2^, ^3^ To safely meet the surgical goal, the laparoscopic approach has become popular,^4^ and robotic surgery has been increasingly used in recent years as a form of minimally invasive surgery (MIS).^5^, ^6^

Bowel reconstruction is a critical part of surgery for CRC. Previously, extracorporeal anastomosis (EA) performed through a small laparotomy was the mainstream procedure.^7^ In recent years, in pursuit of MIS, intracorporeal anastomosis (IA) has been introduced and is gradually becoming popular in laparoscopic and robotic surgery for right‐sided colon cancer (LSRCC).^8^ European randomized controlled trials (RCTs) have demonstrated that IA provides better postoperative outcomes than EA. This is probably due to its smaller intestinal mobilization area, smaller wound size, and avoidance of traumatic traction of organs and vessels^9^, ^10^; however, it has the downside of a longer operative time.^11^

Intracorporeal overlap anastomosis (IOA), designed as an isoperistaltic side‐to‐side anastomosis, is one of the most commonly adopted IA techniques for LSRCC in Japan.^12^, ^13^ Theoretically, compared with other types of IA techniques, IOA has the advantages of structural stability and procedural simplicity. However, the clinical safety of IOA for LSRCC has not yet been fully verified. The IA technique investigated in past RCTs comparing the safety of IA with that of EA was an antiperistaltic anastomosis,^9^, ^10^ which is called functional end‐to‐end anastomosis (FEEA). The few available prospective studies on the safety of IOA included only a small patient population^14^ or a large proportion of benign cases.^15^ Above all, among all complications of LSRCC, there is an inevitable concern regarding the risk of anastomotic leakage (AL). This is because of the unfamiliar and relatively complex technique specific to IOA in the implementation phase. Furthermore, the impact of surgical details, such as the enterotomy closure technique, and clinical factors, such as the learning curve of the surgeon or facility, on the outcome of IOA has not been examined in detail, even in retrospective studies.

Therefore, we conducted the first prospective observational study in Japan to investigate the safety of IOA in LSRCC.

## 2 PATIENTS AND METHODS

### 2.1 Study design

This was a prospective, multicenter, single‐arm observational study. Seven institutions of the Kanagawa Yokohama Colorectal Cancer Research Group (KYCC) participated in this study.

The inclusion criteria were as follows: (1) pathologically proven adenocarcinoma; (2) located in the cecum, ascending colon, or right transverse colon (the anal edge of the tumor was located on the right side of the midline of the body) as confirmed using computed tomography (CT) or enterography; (3) clinically diagnosed based on the Union for International Cancer Control (UICC) TNM classification (8th edition)^16^ as stages I–III; (4) planning to undergo laparoscopic or robotic surgery consisting of ileocecal resection (ICR), right‐hemicolectomy (RHC), or extended right‐hemicolectomy (eRHC), followed by reconstruction or IOA of the ileum and colon; (5) Eastern Cooperative Oncology Group performance status (ECOG PS) 0–2; and (6) ≥20 y old.

The exclusion criteria were as follows: (1) history of neoadjuvant chemotherapy or preoperative radiotherapy to the cancer site; (2) synchronous multiple CRC lesions requiring multiple anastomoses; (3) metachronous multiple CRC lesions requiring resection of the colon or rectum; (4) synchronous resection of other organs planned preoperatively; (5) pregnancy; (6) use of steroids; (7) intraoperatively diagnosed metastasis, such as peritoneal metastasis; and (8) preoperative intestinal obstruction and perforation.

### 2.2 Perioperative treatment

Perioperative treatment varied by the facility. Preoperatively, all facilities adopted mechanical preparation, either with magnesium citrate or picosulfate hydrate. Five of the six facilities also used a chemical preparation consisting of kanamycin sulfate and metronidazole, while one facility did not use any chemical preparation. Cefmetazole was administered as the intra‐ and postoperative antibiotic of choice at all facilities.

### 2.3 Overlap anastomosis and surgical procedure

**FIGURE 1:**
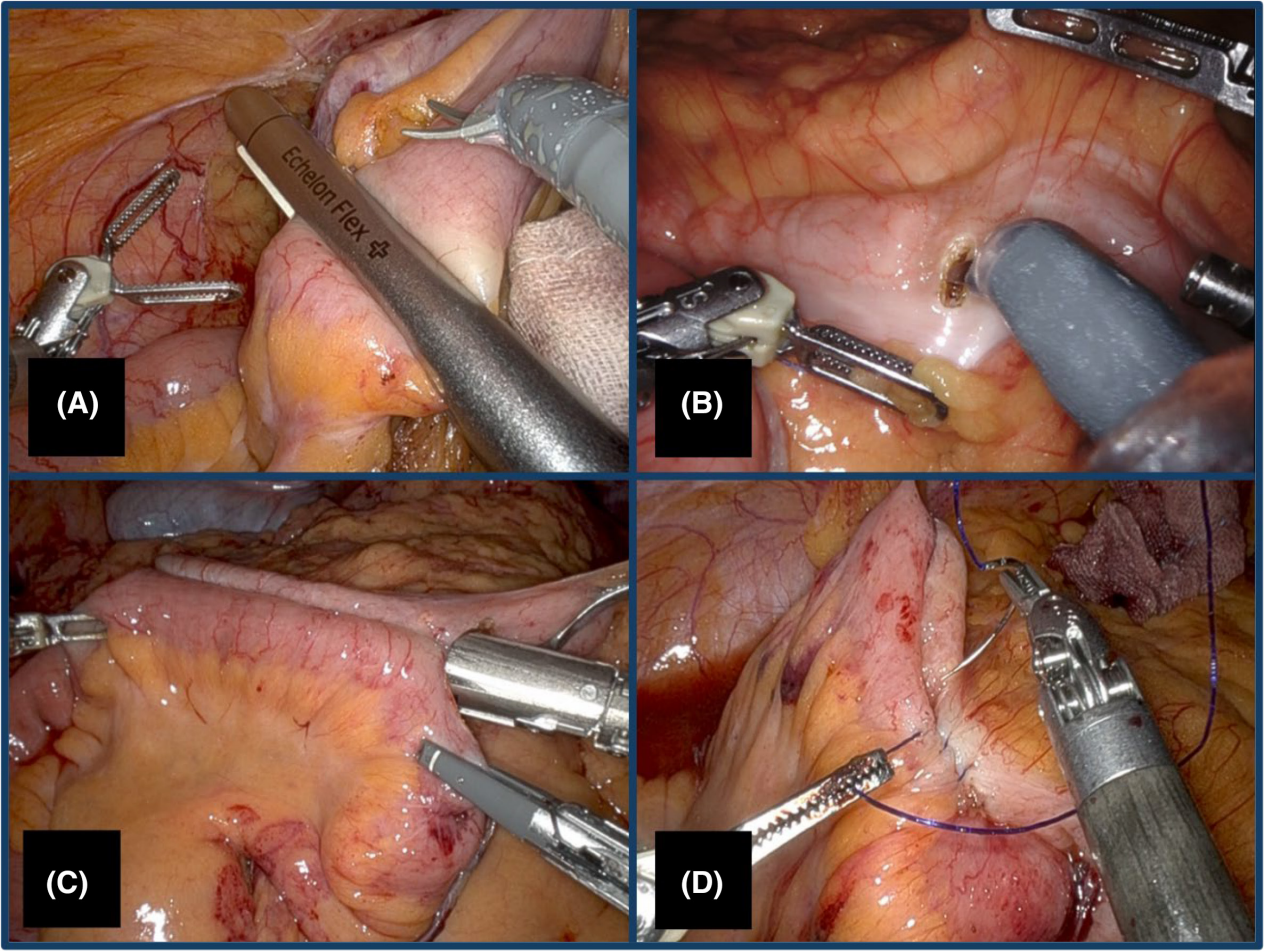
Procedure of intraabdominal overlap anastomosis. (A) Transection of the ileum and colon, (B) enterotomy, (C) isoperistaltic side‐to‐side anastomosis using a stapler, (D) closure of the enterotomy using the suturing technique.

Overlap anastomosis was defined as an isoperistaltic anastomosis of the antimesenteric sides of the ileum and colon. The IOA procedure consisted of four parts: (a) transection of the ileum and colon, (b) enterotomy, (c) side‐to‐side anastomosis using a stapler, and (d) closure of the enterotomy (Figure 1). While introducing the IOA, the fundamental procedures and surgical techniques were shared with all participating facilities in a study meeting, as all facilities were in the implementation phase when this trial started. Subsequently, the surgical techniques were modified and standardized at each facility. The surgical approach and details of the procedures (eg, port setting, order of mobilization of the mesocolon, type of surgical device or stapler used, whether to adopt a reinforcement in the anastomosis, suturing technique for enterotomy closure [SUEC], or stapling technique for enterotomy closure [STEC]) were not restricted but were left to the surgeon's discretion. The extent of lymph node dissection (LND) and length of bowel resection, the oncological concept of which is identical to that of complete mesocolic excision (CME) + central vascular ligation (CVL),^2^ were determined according to the Japanese Society for Cancer of the Colon and Rectum (JSCCR) guidelines 2019 for the treatment of CRC.^17^ D2 LND was defined as LND on the right side of the superior mesenteric vein (SMV) and D3 on the left side. The surgical margins were almost 10 cm from the tumor edge or feeding artery.

### 2.4 Endpoints

The primary outcome of this study was the incidence of AL (Clavien–Dindo classification [C–D] grade ≥3),^18^ and the secondary outcomes were other short‐term and long‐term outcomes. AL was diagnosed if there were feces in the drainage tube or leakage of the contrast agent on enterography. To distinguish between AL and abdominal abscess not due to AL, all patients underwent CT if they had any symptoms or laboratory findings suggestive of AL. If the CT findings showed an abscess that even slightly touched the anastomosis, it was considered as AL and not an abdominal abscess. Regarding short‐term outcomes, the observational period was 30 d after surgery. Intraoperative complications (vascular injury, organ damage, and other events assessed using the Common Terminology Criteria for Adverse Events version 5.0 grade ≥2), postoperative complications (C–D grade ≥2), operative time, amount of blood loss, start of postoperative oral intake, postoperative hospital stay, and readmission were examined. Operative details such as the surgical approach, whether the surgeon or assistant was Endoscopic *Surgical* Skill Qualification System (ESSQS)‐certified, number of cases encountered by each surgeon or at each facility, stapler used, use of a reinforcement sheet, reconstruction time, enterotomy closure technique, and pathological details were prospectively investigated. The time for reconstruction was calculated as the time from performing the enterotomy with the energy device to the removal of the thread or stapler for closure of the enterotomy. The notation of pathological findings was in accordance with the Japanese Classification of Colorectal, Appendiceal, and Anal Carcinoma (9th edition).^19^

### 2.5 Statistical analysis

For the primary endpoint, the frequency and percentage of events and the 95% confidence interval (CI) of Clopper and Pearson were calculated. The median and interquartile range were used to perform an exploratory analysis of the factors that may affect the secondary endpoint of reconstruction time. Confidence intervals for the median were calculated using bootstrap resampling rather than the commonly used Hodges–Lehmann estimator because some sample distributions were asymmetric. The bootstrap resampling was set to 10,000 times. Considering the patient volume at each participating institution, the target sample size that could be recruited in about 2 y was set at 120 cases. The probability of AL occurrence after IA is 4.0%–8.6%, according to previous RCTs.^10^, ^11^ Assuming a conservative 3% probability of the primary endpoint, the probability of this event being observed in one or more subjects was calculated to be 97.4% for the 120 subjects included in this study as a posteriori power. Statistical analysis was conducted using the R software v. 4.2.0 (The R Foundation, Boston, MA, USA).^20^

## 3 RESULTS

### 3.1 Patient characteristics

**FIGURE 2:**
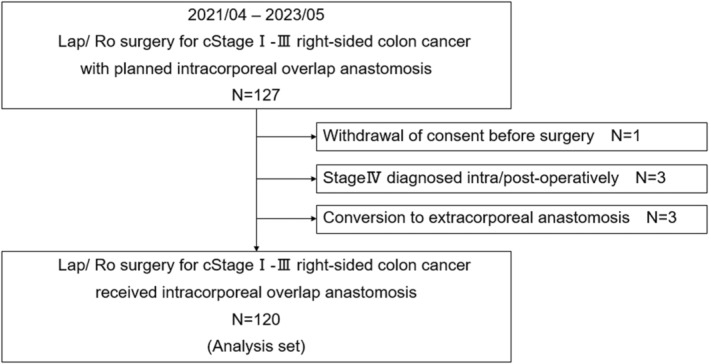
Patient flow diagram. Lap, laparoscopic; Ro, robotic.

The patient flow diagram for this study is shown in Figure 2. In total, 127 patients were enrolled from April 2021 to May 2023. One patient withdrew consent before surgery, and three patients were diagnosed with stage IV disease after the discovery of peritoneal metastasis intraoperatively. Among the remaining 123 patients, three (2.4%) underwent EA for the following reasons: inability to recognize tumor margin inking, exceeding the operative time, and the need to check the blood flow extracorporeally. Finally, 120 patients were included in the analysis.

Patient characteristics are presented in Table 1. The median age and body mass index (BMI) were 76 y and 22.1, respectively. No patients with an EGOG‐PS score >3 were included, although more than 90% of the patients had some comorbidities. A total of 38 (31.7%) patients had previously undergone an abdominal surgery. All patients underwent mechanical preparation. Chemical preparation was administered to 106 (88.3%) patients. Regarding tumor location, 29 (24.2%), 67 (55.8%), and 24 (20.0%) tumors were located in the cecum, ascending colon, and right transverse colon, respectively.

**TABLE 1:** Patient characteristics.

Factor	Category	*N* = 120
Age (y)		76 (37–92)
Sex	Male	57 (47.5%)
	Female	63 (52.5%)
BMI (kg/m^2^)		22.1 (14.9–33.2)
ECOG‐PS	0	89 (74.2%)
	1	25 (20.8%)
	2	6 (5.0%)
ASA‐PS	1	11 (9.2%)
	2	91 (75.8)
	3	18 (15.0%)
Albumin (g/dL)		4.0 (1.9–4.8)
CRP (mg/dL)		0.15 (0.01–12.40)
Previous abdominal surgery		38 (31.7)
Chemical preparation		106 (88.3%)
Mechanical preparation		120 (100%)
Tumor location	Cecum	29 (24.2%)
	Ascending	67 (55.8%)
	Transverse	24 (20.0%)
cStage	I	45 (37.5%)
	II	21 (17.5%)
	III	54 (45.0%)

Abbreviations: ASA‐PS, American Society of Anesthesiology performance status; BMI, body mass index; CRP, C‐reactive protein; ECOG‐PS, Eastern Cooperative Oncology Group performance status.

### 3.2 Operative results

The operative results are summarized in Table 2. A total of 33 surgeons from six facilities participated. Regarding surgical procedures, 59 (49.2%) ICR, 53 (44.1%) RHC, and 8 (6.7%) eRHC procedures were performed. The robotic approach was selected for 16 (13.7%) patients. The median operative time was 257 (118–399) min, and the median blood loss was 3 (0–490) mL. Surgeons in 65 (55.0%) cases and assistants in 94 (78.3%) cases had ESSQS certification. The length of postoperative hospital stay was 7 (4–25) d. No conversion to open surgery or intraoperative adverse events were observed. One patient (0.8%) was readmitted because of worsening renal failure, which was a coexisting disease.

**TABLE 2:** Operative results.

Factor	Category	*N* = 120
Number of facilities		6
Number of surgeons		33
Surgical procedure	ICR	59 (49.2%)
	RHC	53 (44.1%)
	eRHC	8 (6.7%)
Approach	Laparoscopic	104 (86.3%)
	Robotic	16 (13.7%)
LND	D2	1 (0.8%)
	D3	119 (99.2%)
Surgeon with certification of ESSQS		66 (55.0%)
Assistant with certification of ESSQS		94 (78.3%)
Intraoperative lavage (mL)		1000 (500–3000)
Subcutaneous lavage		120 (100%)
Operative time (min)		257 (118–399)
Blood loss (mL)		3 (0–490)
Intraoperative complications		0
Start of oral intake (POD)		3 (1–7)
Conversion to open surgery		0
Readmission		1 (0.8%)
Postoperative hospital stay (d)		7 (4–25)

Abbreviations: eRHC, extended‐right hemicolectomy; ESSQS, Endoscopic Surgical Skill Qualification System by the Japan Society for Endoscopic Surgery; ICR, ileocecal resection; LND, lymph node dissection; POD, postoperative day; RHC, right hemicolectomy.

### 3.3 Postoperative complications

Details of the postoperative complications are presented in Table 3. Postoperative complications (C–D grade ≥ II) were observed in 10 (8.3%) patients. Concerning the primary endpoint of AL (C–D grade ≥ III), only one event (0.83 [95% CI, 0.02–4.56] %) was observed (abscess contiguous with the anastomosis on CT), which was in a case of laparoscopic ICR with SUEC performed by a non‐ESSQS‐certified surgeon. This trend was lower than that observed in previous RCTs.^10^, ^11^ Two patients (one with an abdominal abscess and one with postoperative ileus) required reoperation and were consequently diagnosed with C–D grade IIIb. Another patient diagnosed with C–D grade 3a experienced anastomotic bleeding and underwent endoscopic hemostasis. No complications of C–D grades ≥ IV or surgical site infections of C–D grades ≥ II were observed.

**TABLE 3:** Postoperative complications.

Factor	G II	G IIIa	G IIIb	All grades
Complications	6	2	2	10 (8.3%)
Anastomotic leakage		1		1 (0.8%)
Abdominal abscess	1		1	2 (1.7%)
Ileus	1		1	2 (1.7%)
Anastomotic bleeding		1		1 (0.8%)
Diarrhea	1			1 (0.8%)
Delirium	1			1 (0.8%)
Renal dysfunction	1			1 (0.8%)
Pseudogout	1			1 (0.8%)
Herpes zoster	1			1 (0.8%)

### 3.4 Pathological findings

Table 4 summarizes the pathological findings of all patients. Regarding pathological stage, 43 (35.8%) patients had stage I, 44 (36.7%) had stage II, and 33 (27.5%) had stage III cancer. The median lengths of the proximal and distal margins were 12.5 and 11.7 cm, respectively. R0 curative resection was accomplished in all cases, although two patients with T4b CRC (invasion of the abdominal wall) underwent combined resection of the abdominal wall.

**TABLE 4:** Pathological findings.

Factor	Category	*N* = 120
Histopathological type	Differentiated type	111 (92.5%)
	Undifferentiated type	9 (7.5%)
pT	T1	31 (25.8%)
	T2	15 (12.5%)
	T3	54 (45.0%)
	T4	20 (16.7%)
pN	N0	87 (72.5%)
	N1	22 (18.3%)
	N2	11 (9.2%)
	N3	0
pStage	I	43 (35.8%)
	II	44 (36.7%)
	III	33 (27.5%)
Tumor diameter (mm)		40 (6–150)
LN harvested		24 (3–76)
Length of PM (cm)		12.5 (5.0–40.0)
Length of DM (cm)		11.7 (5.0–26.0)
Curability	R0	120 (100%)

Abbreviations: DM, distal margin; LN, lymph node; PM, proximal margin; pN, pathological N; pStage, pathological stage; pT, pathological T.

### 3.5 Details of reconstruction

Table 5 summarizes the details of the IOA procedure. Linear staplers were used for bowel division and side‐to‐side anastomosis in all cases. For anastomosis, stapling with absorbent sutures for reinforcement was performed in 10 (8.3%) patients. Regarding the techniques of enterotomy closure, the suturing technique was adopted in 80 cases (66.7%) and the stapling technique in 40 cases (33.3%). Consequently, the median number of linear staplers required was three and that of barbed sutures was two. To accomplish reconstruction using IOA, a median time of 32 min was required.

**TABLE 5:** Details of bowel reconstruction by IOA.

Factor	Category	*N* = 120
Absorbent suture reinforcement		14 (11.7%)
Closure of enterotomy	STEC	40 (33.3%)
	SUEC	80 (66.7%)
Number of linear staplers		3 (3–4)
Number of barbed sutures		2 (0–2)
Reconstruction time (min)		32 (6–83)

Abbreviations: IOA, intracorporeal overlap anastomosis; STEC, stapling technique of enterotomy closure; SUEC, suturing technique of enterotomy closure.

The effects of clinical factors and surgical details on IOA reconstruction time are listed in Table 6. STEC required a shorter reconstruction time than SUEC (20.5 [95% CI, 18.0–23.0] min vs 36.5 [95% CI, 35.0–39.0] min). With regard to surgical experience, experience of 30 cases in the facility was associated with a shorter reconstruction time, while the surgeon's experience and ESSQS certification status did not have a positive impact.

**TABLE 6:** Effect of clinical and surgical factors on the reconstruction time.

Factor	*N*	Median (Q1–Q3)	95% CI for the median	95% CI for the difference in medians
Age (y)				
<75	48	31.0 (18.8–40.0)	28.0–35.0	−5.0 to 7.0
75≤	72	32.0 (25.0–40.2)	29.5–35.0	
Sex				
Female	63	30.0 (22.0–36.5)	27.0–34.0	−1.0 to 10.0
Male	57	35.0 (23.0–42.0)	31.0–38.0	
BMI (kg/m^2^)				
<25	95	34.0 (23.0–41.0)	30.0–35.5	−11.0 to 2.0
25≤	25	29.0 (20.0–35.0)	23.0–35.0	
Albumin (g/dL)				
<4.0	58	32.5 (23.5–40.0)	29.0–37.0	−6.5 to 5.0
4.0≤	62	32.0 (20.2–40.5)	29.0–35.0	
CRP (mg/dL)				
<0.3	78	30.5 (23.0–38.0)	29.0–34.0	−2.0 to 9.0
0.3≤	42	35.0 (23.0–42.0)	29.0–40.0	
Previous abdominal surgery				
No	82	30.0 (20.0–39.5)	28.0–34.0	−1.0 to 9.5
Yes	38	35.5 (28.2–40.8)	31.0–39.0	
Surgery				
ICR	59	32.0 (23.0–39.5)	29.0–36.0	−6.0 to 4.5
RHC/eRHC	61	32.0 (21.0–41.0)	28.5–35.0	
Approach				
Lap	103	32.0 (21.5–39.5)	29.0–35.0	−4.0 to 10.0
Ro	17	35.0 (29.0–41.0)	29.0–41.0	
Enterotomy closure				
STEC	40	20.5 (18.0–25.2)	18.0–23.0	12.0 to 20.0
SUEC	80	36.5 (30.8–42.0)	35.0–39.0	
Surgeon ESSQS				
No	54	31.0 (20.2–42.0)	25.5–37.0	−5.0 to 8.0
Yes	66	32.0 (24.0–38.8)	30.0–35.0	
Surgeon experience				
<5	59	30.0 (21.0–40.5)	26.0–35.0	−3.0 to 8.0
5≤	61	34.0 (23.0–39.0)	30.0–35.5	
Surgeon experience				
<10	90	32.0 (21.0–39.8)	28.0–34.5	−3.0 to 7.5
10≤	30	35.0 (28.2–41.0)	29.0–39.0	
Surgeon experience				
<15	113	32.0 (21.0–40.0)	29.0–35.0	−6.0 to 9.0
15≤	7	30.0 (29.0–38.0)	28.0–41.0	
Facility experience				
<10	18	35.0 (23.8–39.0)	25.5–39.0	−9.0 to 7.0
10≤	102	32.0 (22.2–40.0)	29.0–35.0	
Facility experience				
<20	49	34.0 (23.0–39.0)	29.0–37.0	−7.0 to 3.5
20≤	71	32.0 (22.0–41.0)	29.0–35.0	
Facility experience				
<30	81	35.0 (28.0–41.0)	32.0–37.5	−15.0 to −4.0
30≤	39	25.0 (18.5–34.5)	20.0–30.0	

Abbreviations: BMI, body mass index; CRP, C‐reactive protein; eRHC, extended‐right hemicolectomy; ESSQS, Endoscopic Surgical Skill Qualification System by the Japan Society for Endoscopic Surgery; ICR, ileocecal resection; RHC, right hemicolectomy; STEC, stapling technique of enterotomy closure; SUEC, suturing technique of enterotomy closure.

## 4 DISCUSSION

This study aimed to prospectively investigate the safety of the IOA technique for LSRCC and, to the best of our knowledge, is the first prospective study in Japan. We confirmed that the incidence of AL was acceptable and that the IOA technique could be feasibly implemented for LSRCC reconstruction. Furthermore, our exploratory analysis suggested that using STEC for enterotomy closure and sufficient experience of the facility could potentially shorten reconstruction and total operative times.

In the present study the incidence of AL, which was the primary endpoint, was 0.8%. In previous RCTs, the incidence of AL following LSRCC was 4.0%–8.6% for IA with FEEA and 2.9%–7.8% for EA.^9^, ^10^ However, in other studies the incidence of AL after LSRCC was reported to be lower. In retrospective cohort studies conducted by Teramura et al and Liao et al to compare the short‐term results of EA and IA, the incidence rates of AL after IA were reported to be 1.0% and 1.1%, respectively.^12^, ^21^ However, these studies may have underestimated the incidence of AL because of their retrospective nature. Additionally, even in prospective studies, AL may have been overlooked as an adverse event. In particular, when discussing IA complications it is important to distinguish AL from abdominal abscesses. In the present study, we clearly defined AL based on CT findings and distinguished it from an abdominal abscess. Therefore, our results can be considered reliable.

Several aspects of the surgical procedure, such as the approach, techniques, configuration, and surgical devices used, can result in heterogeneity of bowel reconstruction (eg, Ro vs Lap, IA vs EA, antiperistaltic technique vs isoperistaltic technique, STEC vs SUEC). In terms of the direction of peristalsis, available evidence shows equivalent safety of antiperistaltic and isoperistaltic anastomosis.^22^ However, the configuration of isoperistaltic anastomosis has potential merits in that it has no cloches, which is the structurally fragile part and can be a risk factor for AL, and a smaller waste lumen, which can be a blind end, is also present.^23^ However, under conditions of intracorporeal manipulation the features of isoperistaltic methods, including easier alignment of the anastomotic axis with the bowel axis, likely have more advantages than those of FEEA.^15^ There are no studies comparing the safety of IOA and hemi‐FEEA, which is called the delta anastomosis.^24^, ^25^ Theoretically, compared with the delta technique, the overlap technique has a low risk of penetrating the bowel wall with the stapler tip while making the anastomosis. Furthermore, in IOA the complete contra‐mesenteric side is anastomosed, resulting in a lower risk of pinching of the mesial adipose tissue. This can lead to an adequate thickness of the stapled tissue in the anastomosis, less fatty tissues obstructing the field of view, and precise recognition of the bowel wall during enterotomy closure, especially under SUEC. These features may contribute to the lower incidence of AL in IOA, although the delta technique reportedly has a shorter operative time.^25^

The incidence of abdominal abscesses is of great concern for patients with IA. In an ad‐hoc analysis of a prospective study, Sun et al reported that the incidence of abdominal abscess following IOA in LSRCC was as high as 9.2%.^26^ This was significantly higher than that following EA. We encountered only two cases (1.7%) of abdominal abscess. One case was suspected to have been caused by necrosis of the omentum due to impaired blood flow, as suggested by an enhanced CT scan. In the other case, the abscess was localized just below the midline wound, where antiadhesive materials were used, with no abnormal findings around the anastomotic site. In our study, owing to the exclusion of patients with preoperative obstruction and perforation, almost 90% of the patients were successfully treated with mechanical preparation, as well as the preoperative prophylactic antibiotics kanamycin and metronidazole. Although there is no strong evidence to support the use of chemical preparation with these agents, this may have contributed to the lower incidence of abdominal abscess.

A meta‐analysis raised the important issue that the operative time of IA is longer than that of EA.^11^, ^15^ In the current study, the median operative time was 257 min, which was longer than that for LSRCC, according to the national clinical database in Japan.^27^ Prolonged reconstruction time due to the technically demanding procedure of IA^24^ is reportedly the main reason,^12^ and in the current study the median reconstruction time was 32 min. To date, few studies have focused on the reconstruction time for IA. The results of our exploratory analysis suggested two factors for reducing reconstruction time. First, STEC should be used. With the advent of barbed sutures, the time required for SUEC has been greatly reduced.^13^, ^28^ However, the reconstruction time was 12 min shorter in cases with STEC; nevertheless, barbed sutures were used in all cases with SUEC.^24^, ^29^ Second, the reconstruction time was shorter when 30 patients were treated at a single institution. In a previous report, the skill and experience of the surgeon reduced the operative time of laparoscopic surgery.^30^ However, our results suggest that neither the surgeon's experience nor the ESSQS, but the facility experience has a crucial impact on the learning curve for bowel reconstruction. This implies that the bowel reconstruction procedure requires more complex coordination of the surgical team than the CME and CVL procedures. Considering that all facilities included in this study were in the implementation phase, further reduction in reconstruction time, followed by operative time, may be expected in the future.

In the current study the IOA technique required a total of three staplers to accomplish the reconstruction, mainly because SUEC was used in most cases. However, it is noteworthy that when STEC was adopted, the enterotomy could be closed with a single shot of a linear stapler in all cases, whereas other techniques for enterotomy closure have been reported to sometimes require multiple shots for enterotomy closure.^24^ An adequate thickness of the resected tissue and good surgical view under closing enterotomy, which are the advantages of IOA, may provide the shortest stapler‐cut line and reduce the number of staplers required. IOA could be the most cost‐effective technique among all IA types.

This study has some limitations. First, this study was not designed in a comparative setting, and the results of an RCT on IA were selected as the reference. To clarify the real advantages of the IOA technique, clinical trials designed to compare IOA with delta or FEEA techniques are required. Second, perioperative treatment was not unified and could have influenced the results observed. Third, this study did not have any stipulations for surgeons. However, this is in line with actual clinical practice and shows that IOA can be safely performed even by novice surgeons. Fourth, this study excluded patients with stage 4 disease and those using steroidal drugs. These are reported to be risk factors for AL and abdominal abscesses; therefore, the complication rates may be higher in clinical practice. Fifth, robot‐assisted and laparoscopic approaches were analyzed together, resulting in heterogeneity in the anastomoses. Survival data (3‐year overall survival) and detailed information on recurrence were collected as secondary endpoints in this study.

## 5 CONCLUSION

IOA is a feasible method for reconstruction in LSRCC and can be safely performed even in the implementation phase, regardless of the surgeon's experience.

## AUTHOR CONTRIBUTIONS

Keisuke Kazama: conceptualization, methodology, writing of draft; review and editing. Masakatsu Numata: conceptualization, methodology, data management, review. Mushiake Hiroyuki: methodology and data management. Nobuhiro Sugano: methodology and data management. Teni Godai: methodology and data management. Akio Higuchi: methodology and data management. Tetsushi Ishiguro: methodology and data management. Yosuke Atsumi: methodology and data management. Satoru Shinoda: formal analysis. Aya Saito: conceptualization and review. All authors read and approved the final article.

## FUNDING INFORMATION

None declared.

## CONFLICT OF INTEREST STATEMENT

The authors declare no conflicts of interest.

## ETHICS STATEMENT

Approval of the research protocol: The protocol for this research project has been approved by a suitably constituted Ethics Committee of the institution, and it conforms to the provisions of the Declaration of Helsinki. Committee of Yokohama City University and each participating hospital, Approval No. B230200083.

Informed Consent: All informed consent was obtained from the subjects.

Registry and the Registration No. of the study/trial: This study was registered with the University of Hospital Medical Information Network Clinical Trials Registry (UMIN‐CTR) in Japan (UMIN 000043987).

Animal Studies: N/A.

## Data Availability

The collected data or related documents will not be made available to others.

## References

[1] Bray F , Ferlay J , Soerjomataram I , Siegel RL , Torre LA , Jemal A . Global cancer statistics 2018: GLOBOCAN estimates of incidence and mortality worldwide for 36 cancers in 185 countries. CA Cancer J Clin. 2018;68(6):394–424. 30207593 10.3322/caac.21492

[2] Hohenberger W , Weber K , Matzel K , Papadopoulos T , Merkel S . Standardized surgery for colonic cancer: complete mesocolic excision and central ligation–technical notes and outcome. Colorectal Dis. 2009;11(4):354–364; discussion 364–365. 19016817 10.1111/j.1463-1318.2008.01735.x

[3] Veldkamp R , Kuhry E , Hop WC , Jeekel J , Kazemier G , Bonjer HJ , et al. Laparoscopic surgery versus open surgery for colon cancer: short‐term outcomes of a randomised trial. Lancet Oncol. 2005;6(7):477–484. 15992696 10.1016/S1470-2045(05)70221-7

[4] Kitano S , Inomata M , Mizusawa J , Katayama H , Watanabe M , Yamamoto S , et al. Survival outcomes following laparoscopic versus open D3 dissection for stage II or III colon cancer (JCOG0404): a phase 3, randomised controlled trial. Lancet Gastroenterol Hepatol. 2017;2(4):261–268. 28404155 10.1016/S2468-1253(16)30207-2

[5] Numata M , Watanabe J , Ishibe A , Ozawa M , Suwa Y , Kazama K , et al. Surgical outcomes of a prospective, phase 2 trial of robotic surgery for resectable right‐sided colon cancer (the ROBOCOLO trial). Ann Gastroenterol Surg. 2024;8(1):80–87. 38250687 10.1002/ags3.12718 PMC10797943

[6] Yamauchi S , Shiomi A , Matsuda C , Takemasa I , Hanai T , Uemura M , et al. Robotic‐assisted colectomy for right‐sided colon cancer: short‐term surgical outcomes of a multi‐institutional prospective cohort study in Japan. Ann Gastroenterol Surg. 2023;7(6):932–939. 37927933 10.1002/ags3.12694 PMC10623957

[7] Würtz HJ , Bundgaard L , Rahr HB , Frostberg E . Anastomosis technique and leakage rates in minimally invasive surgery for right‐sided colon cancer. A retrospective national cohort study. Int J Colorectal Dis. 2022;37(3):701–708. 35150297 10.1007/s00384-022-04107-9

[8] Gómez Ruiz M , Espin‐Basany E , Spinelli A , Cagigas Fernández C , Bollo Rodriguez J , María Enriquez Navascués J , et al. Early outcomes from the minimally invasive right colectomy anastomosis study (MIRCAST). Br J Surg. 2023;110(12):1906. 10.1093/bjs/znad077 37289913 PMC10416692

[9] Bollo J , Turrado V , Rabal A , Carrillo E , Gich I , Martinez MC , et al. Randomized clinical trial of intracorporeal versus extracorporeal anastomosis in laparoscopic right colectomy (IEA trial). Br J Surg. 2020;107(4):364–372. 31846067 10.1002/bjs.11389

[10] Allaix ME , Degiuli M , Bonino MA , Arezzo A , Mistrangelo M , Passera R , et al. Intracorporeal or extracorporeal ileocolic anastomosis after laparoscopic right colectomy: a double‐blinded randomized controlled trial. Ann Surg. 2019;270(5):762–767. 31592811 10.1097/SLA.0000000000003519

[11] Zhang T , Sun Y , Mao W . Meta‐analysis of randomized controlled trials comparing intracorporeal versus extracorporeal anastomosis in minimally invasive right hemicolectomy: upgrading the level of evidence. Int J Colorectal Dis. 2023;38(1):147. 37248431 10.1007/s00384-023-04445-2

[12] Teramura K , Kitaguchi D , Matsuoka H , Hasegawa H , Ikeda K , Tsukada Y , et al. Short‐term outcomes following intracorporeal vs extracorporeal anastomosis after laparoscopic right and left‐sided colectomy: a propensity score‐matched study. Int J Surg. 2023;109(8):2214–2219. 37222668 10.1097/JS9.0000000000000485 PMC10442079

[13] Hamamoto H , Okuda J , Izuhara K , Ishii M , Osumi W , Masubuchi S , et al. Closure of enterotomy after side‐to‐side ileocolic anastomosis with two barbed sutures in totally laparoscopic colectomy for right‐sided colon cancer. Surg Today. 2021;51(3):457–461. 32780157 10.1007/s00595-020-02108-1 PMC7892497

[14] Sorgato N , Mammano E , Contardo T , Vittadello F , Sarzo G , Morpurgo E . Right colectomy with intracorporeal anastomosis for cancer: a prospective comparison between robotics and laparoscopy. J Robot Surg. 2022;16(3):655–663. 34368911 10.1007/s11701-021-01290-9

[15] Cleary RK , Silviera M , Reidy TJ , McCormick J , Johnson CS , Sylla P , et al. Intracorporeal and extracorporeal anastomosis for robotic‐assisted and laparoscopic right colectomy: short‐term outcomes of a multi‐center prospective trial. Surg Endosc. 2022;36(6):4349–4358. 34724580 10.1007/s00464-021-08780-9 PMC9085698

[16] James DB , Mary KG , Christian W . TNM classification of malignanttumours. 8th ed. New York: Wiley Blackwell; 2017.

[17] Hashiguchi Y , Muro K , Saito Y , Ito Y , Ajioka Y , Hamaguchi T , et al. Japanese Society for Cancer of the Colon and Rectum (JSCCR) guidelines 2019 for the treatment of colorectal cancer. Int J Clin Oncol. 2020;25(1):1–42. 31203527 10.1007/s10147-019-01485-z PMC6946738

[18] Dindo D , Demartines N , Clavien PA . Classification of surgical complications: a new proposal with evaluation in a cohort of 6336 patients and results of a survey. Ann Surg. 2004;240(2):205–213. 15273542 10.1097/01.sla.0000133083.54934.ae PMC1360123

[19] Japanese Society for Cancer of the Colon and Rectum . Japanese classification of colorectal, appendiceal, and anal carcinoma: the 3rd English edition [secondary publication]. J Anus Rectum Colon. 2019;3(4):175–195. 31768468 10.23922/jarc.2019-018 PMC6845287

[20] R Core Team . R: a language and environment for statistical computing. Vienna, Austria: R Foundation for Statistical Computing; 2023.

[21] Liao CK , Chern YJ , Lin YC , Hsu YJ , Chiang JM , Tsai WS , et al. Short‐ and medium‐term outcomes of intracorporeal versus extracorporeal anastomosis in laparoscopic right colectomy: a propensity score‐matched study. World J Surg Oncol. 2021;19(1):6. 33397412 10.1186/s12957-020-02112-2 PMC7783968

[22] Matsuda A , Miyashita M , Matsumoto S , Sakurazawa N , Takahashi G , Yamada M , et al. Isoperistaltic versus antiperistaltic stapled side‐to‐side anastomosis for colon cancer surgery: a randomized controlled trial. J Surg Res. 2015;196(1):107–112. 25818976 10.1016/j.jss.2015.02.059

[23] Ibañez N , Abrisqueta J , Luján J , Hernández Q , Parrilla P . Isoperistaltic versus antiperistaltic side‐to‐side anastomosis after right laparoscopic hemicolectomy for cancer (ISOVANTI) trial: study protocol for a randomised clinical trial. Int J Colorectal Dis. 2017;32(9):1349–1356. 28634703 10.1007/s00384-017-2840-6

[24] Watanabe J , Ishibe A , Takei S , Suwa Y , Suwa H , Endo I . A new intracorporeal suture‐less stapled end‐to‐end anastomosis in laparoscopic left‐colectomy for colon cancer – a video vignette. Colorectal Dis. 2020;22(11):1803–1804. 32620045 10.1111/codi.15232

[25] Zhou HT , Wang P , Liang JW , Su H , Zhou ZX . Short‐term outcomes of overlapped delta‐shaped anastomosis, an innovative intracorporeal anastomosis technique, in totally laparoscopic colectomy for colon cancer. World J Gastroenterol. 2017;23(36):6726–6732. 29085217 10.3748/wjg.v23.i36.6726 PMC5643293

[26] Sun R , Zhang Y , Feng B , Su X , Sun Y , Xu L , et al. Intracorporeal anastomosis versus extracorporeal anastomosis in laparoscopic right colectomy: an observational cohort study. World J Surg. 2023;47(3):785–795. 36635607 10.1007/s00268-022-06834-0

[27] Matsuda T , Endo H , Inomata M , Hasegawa H , Kumamaru H , Miyata H , et al. Clinical outcome of laparoscopic vs open right hemicolectomy for colon cancer: a propensity score matching analysis of the Japanese National Clinical Database. Ann Gastroenterol Surg. 2020;4(6):693–700. 33319160 10.1002/ags3.12381 PMC7726676

[28] Bautista T , Shabbir A , Rao J , So J , Kono K , Durai P . Enterotomy closure using knotless and barbed suture in laparoscopic upper gastrointestinal surgeries. Surg Endosc. 2016;30(4):1699–1703. 10.1007/s00464-015-4395-3 26173547

[29] Milone M , Elmore U , Allaix ME , Bianchi PP , Biondi A , Boni L , et al. Fashioning enterotomy closure after totally laparoscopic ileocolic anastomosis for right colon cancer: a multicenter experience. Surg Endosc. 2020;34(2):557–563. 31011862 10.1007/s00464-019-06796-w

[30] Ichikawa N , Homma S , Hida K , Akagi T , Kamada Y , Yamaguchi T , et al. Impact of endoscopic surgical skill qualification on laparoscopic resections for rectal cancer in Japan: the EnSSURE study. Ann Surg Open. 2022;3(2):e160. 37601611 10.1097/AS9.0000000000000160 PMC10431478

